# Behavior of Biochar-Modified Cementitious Composites Exposed to High Temperatures

**DOI:** 10.3390/ma14185414

**Published:** 2021-09-18

**Authors:** Xu Yang, Run-Sheng Lin, Yi Han, Xiao-Yong Wang

**Affiliations:** 1 Department of Architectural Engineering, Kangwon National University, Chuncheon-si 24341, Korea; yangxu@kangwon.ac.kr (X.Y.); linrunsheng@kangwon.ac.kr (R.-S.L.); 2 Department of Integrated Energy and Infra System, Kangwon National University, Chuncheon-si 24341, Korea; hanyii@kangwon.ac.kr

**Keywords:** biochar, high temperature, residual strength, meso crack, microstructures

## Abstract

In this study, the effect of biochar on the high temperature resistance of cementitious paste was investigated using multiple experimental methods. The weight loss, cracks, residual compressive strength, and ultrasonic pulse velocity (UPV) of biochar cementitious paste with 2% and 5% biochar exposed to 300, 550 and 900 °C were measured. The products and microstructures of biochar cementitious paste exposed to high temperatures were analyzed by X-ray diffraction, Fourier transform infrared spectroscopy, thermogravimetric analysis, and scanning electron microscopy. The results showed that the cracks of specimens exposed to high temperatures decreased with increasing biochar content. The addition of 2% and 5% biochar increased the residual compressive strength of the specimens exposed to 300 °C and the relative residual compressive strength at 550 °C. As the exposure temperature increased, the addition of biochar compensated for the decreasing ultrasonic pulse velocity. The addition of biochar contributed to the release of free water and bound water, and reduced the vapor pressure of the specimen. The addition of biochar did not change the types of functional groups and crystalline phases of the products of cementitious materials exposed to high temperatures. Biochar particles were difficult to observe at 900 °C in scanning electron microscopy images. In summary, because biochar has internal pores, it can improve the high-temperature resistance of cement paste.

## 1. Introduction

The properties of building materials exposed to high temperatures are directly related to the magnitude of risk faced by the building in the event of a fire. High-temperature damage to concrete includes cracks, strength loss, spalling, etc. [1]. The main causes of damage to cementitious materials exposed to high temperatures can be categorized into two areas: physical changes, such as cracks caused by vapor pressure; and chemical changes, mainly decomposition of cementitious hydration products that support strength.

Previous studies showed that suitable fillers or additives contribute to the performance of cementitious materials exposed to high temperatures and improve the high-temperature resistance of concrete. For instance, the inclusion of volcanic ash, fly ash, etc., as pozzolanic materials in cementitious materials, resulted in lower thermal damage due to their pozzolanic effect [2,3]. Irshidat et al. [4] investigated the thermal properties of nanoclay-modified mortars, and their results showed that the addition of the nanoclay had a positive effect on the residual strength when exposed to higher temperatures. In addition, the creation of a pore network in the material to reduce the pore pressure when exposed to high temperatures was established as an effective method to reduce thermal damage to cementitious materials. Kalifa et al. [5] reported that the melting of polypropylene (PP) fibers during heating to form gas pathways contributes to the reduction in pore pressure to improve the thermal properties of high-performance concrete (HPC), and provided a threshold value (2 kg/m^3^) for the addition of PP fibers to prevent spalling in HPC. Biochar, as a porous material that pyrolyzes at high temperatures under anaerobic conditions, is widely used in construction materials. Some previous studies showed that biochar is excellent in improving the mechanical properties and durability of cementitious composites [6,7].

At present, few particular studies discuss the thermal performance of biochar in cementitious materials exposed to high temperatures. Gupta et al. [8] found that concrete containing biochar exposed to 300 and 550 °C showed higher residual compressive strength than plain concrete and concrete containing silica fume. Gupta and Kua [9] reported that rice husk biochar contributed to reducing internal damage and increasing the residual compressive strength of silica fume and cenosphere-modified mortar exposed to 450 °C. Although the performance of cementitious paste under high temperature has been well studied, the study on the high temperature properties of cementitious composites using biochar as a partial replacement for cement is relatively limited. To fill this knowledge gap, we performed a series of experimental studies about the performance of cement–biochar blends under various high temperatures. The novelties of this study are summarized as follows: (1) There are no documented studies on the properties of biochar cementitious composites exposed to higher temperatures (550–900 °C), which occur in reality. Therefore, we studied the properties of biochar cementitious materials exposed to 300, 550 and 900 °C compared to 20 °C. (2) At high temperatures, meso cracks are generated in concrete, and various properties of concrete are closely related to meso cracks. The previous studies on biochar cementitious materials did not investigate meso cracks, so we studied meso cracks at different temperatures with an optical microscope. (3) High temperature damages concrete at the macro, meso and micro levels. Previous studies focused on a single level, and the macro, meso and micro relationships are unclear. In this study, the macro, meso and micro properties of biochar cementitious composites at high temperatures were systematically investigated using various experimental methods, and the relationships between the interactions at various levels were clarified.

The research impacts of this study are summarized as follows: (1) clarifies the mechanism of biochar to improve the high temperature performance of cementitious composites. (2) provides an experimental basis for further research on the high temperature performance of biochar blended cementitious composites. (3) supplies a theoretical basis for the application of biochar blended cementitious composites in engineering practice.

## 2. Materials and Methods

### 2.1. Materials and Mix Design

**Figure 1:**
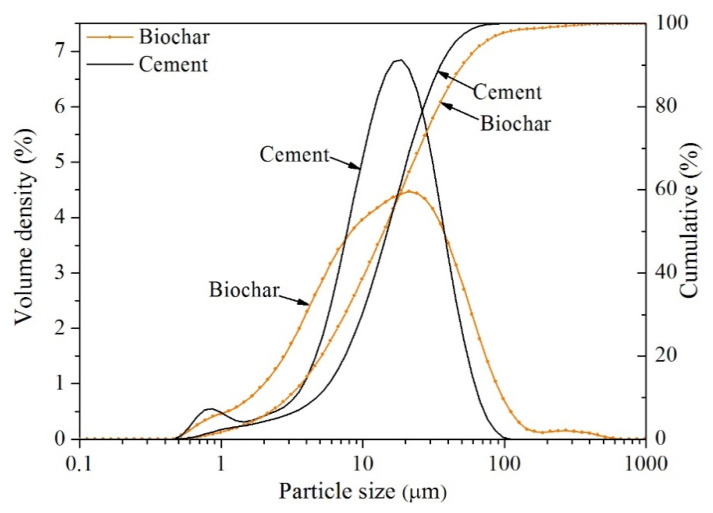
Particle size distributions of biochar and cement.

**Figure 2:**
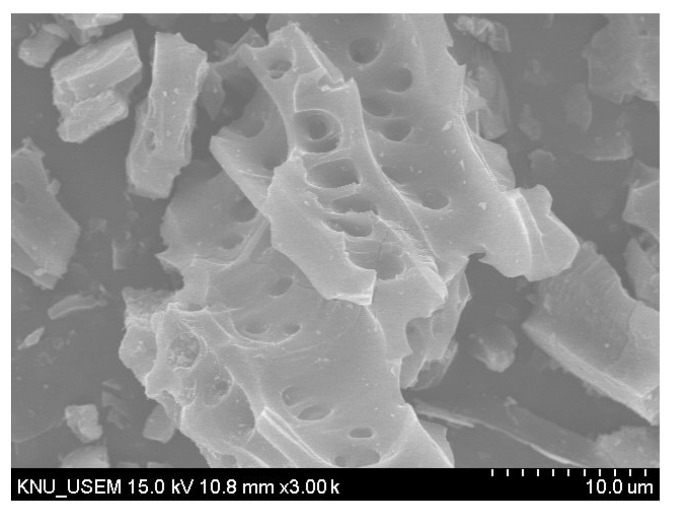
SEM image of biochar.

Type I (ISO 9001/14001) Portland cement and commercial biochar, provided by South Korea Sungshin Cement Co., Ltd. (Seoul, Korea) and Yoogi IND Co., Ltd. (Daegu, Korea), were used in this study. The biochar was ground into powder by a grinder. The particle size of the cement and biochar powder was measured with a laser diffraction analyzer (Mastersizer 3000), and the results are shown in Figure 1. The particle size distribution ranges of biochar and cement were 0.523–586 μm and 0.523–98.1 μm, respectively. D50 corresponds to 50% of the total accumulation of particles, and the D50 of biochar and cement were 15.9 and 16.9 μm, respectively. The chemical composition of cement and biochar was measured with an X-ray fluorescence analyzer (ZSX Primus II) (Rigaku, Austin, TX, USA), and the results are shown in Table 1. The porous microscopic image of biochar powder was characterized by scanning electron microscopy (SEM) (Hitachi S-4800) (Hitachi, Tokyo, Japan), as shown in Figure 2. In this study, all specimens were a paste with a water-to-cement ratio of 0.5 and biochar replacement levels of 2% and 5% of the cement mass. The mix design is listed in Table 2.

### 2.2. Methods

According to the mix design in Table 2, the biochar was first added and mixed with water and dispersed in the ultrasonic instrument (WUC-A22H, DAIHAN Scientific, Gang-won, Korea) for 2 min. Then, the cement was added to the biochar, mixed with water in a HOBART blender container (HL200, HOBART, Glenview, IL, USA), and mixed at low speed for 2 min and high speed for 3 min. Finally, the mixed paste was poured into a 50 × 50 × 50 mm^3^ cube mold, covered using a film, and demolded after 24 h. The specimens were wrapped with film, sealed, and cured in a constant-temperature chamber (IR-250, DAIHAN Scientific, Gang-won, Korea) at 20 °C for 28 days. The high-temperature resistance test of the hardened cement paste specimens at the age of 28 days was performed in a muffle furnace (Nabertherm L15/12/P330, Nabertherm, Germany) with a heating rate of 5 °C/min, at 300, 550, and 900 °C for 2 h. The specimens were cooled down naturally in the muffle furnace. Table 3 summarizes the experimental methods and specimen types used to characterize the performance of the specimens in this study.

#### 2.2.1. Macro Performance Characterization Methods

The specimens (50 × 50 × 50 mm^3^) were weighed before and after exposure to the three high temperatures to calculate the weight loss percentage. The apparent characteristics of the specimens exposed to 20 °C and high temperatures were observed with an optical microscope (LEICA Z16 APO) (LEICA, Wetzlar, Germany) at a magnification of 61. A digital motorized compression tester (SGB-F-200D) (Shin Gang, Seoul, Korea) was used to test the compressive strength at 20 °C and the residual compressive strength of cubic specimens exposed to high temperatures according to American Society for Testing and Materials (ASTM) C109 [10]. According to ASTM C597 [11], an ultrasonic velocity tester (Pundit Lab) (Proceq Company, Schwerzenbach, Switzerland) was used to test the ultrasonic pulse velocity (UPV) of specimens at 20, 300, 550 and 900 °C at a frequency of 54 Hz. The final results of weight loss, compressive strength, and UPV are presented as the average of three specimens.

#### 2.2.2. Micro Characterization Methods

CC, CB2 and CB5 specimens, which were cured at 20 °C for 28 days, were cored, wet-milled with isopropyl alcohol, and vacuum-treated to stop hydration, and then thermogravimetric (TG) tests were performed with a thermal analyzer (SETARAM FR/W5 DTA rod) (SETARAM, Lyon, France). The heating rate and temperature range were 10 °C/min and 20–900 °C, respectively, with an inert gas nitrogen purge. Fourier transform infrared spectroscopy (FTIR) (Spectrum IR FTIR spectrometer, PerkinElmer, Waltham, MA, USA) and X-ray diffraction (XRD) (X’Pert PRO MPD, PANalytical, Almelo, Netherlands) experiments were performed on all specimens (20, 300, 550 and 900 °C), and the sampling methods were the same as those mentioned above for the TG tests. The FTIR spectrum was scanned in the range of 400–4000 cm^−1^. The XRD scan increments were 0.013° in the range of 2θ 5~50° (λ = 1.5406 Å, 40 kV, and 30 Ma), with a cumulative time of 8.67 s per step. SEM images of the CC and CB5 specimens were analyzed by a Hitachi S-4800 Ultra-High-Resolution Scanning Electron Microscope (UHR-SEM) (Hitachi, Tokyo, Japan). The surface pretreatment of samples was performed using a Leica EM ACE600 Specimen Coater (LEICA, Wetzlar, Germany).

At present, the study about the high temperature performance of the biochar-added cementitious materials is very limited, so we conducted a series of studies on paste samples using different substitution ratios at different temperatures. Compared to paste samples, concrete samples are much more complex due to the addition of fine and coarse aggregate. Hence when the samples are concrete, a large number of samples should be used. In the future, we intend to perform more studies on concrete with a large number of samples.

## 3. Results and Discussion

### 3.1. Optical Microscopic Image

Table 4 depicts the optical microscope images of the surfaces of all specimens under the same exposure and the same magnification. This is one of the relatively straightforward methods used to judge the high-temperature resistance of cementitious materials by observing the appearance of the specimen. Appearance judgments include changes in color, cracks, explosive spalling, etc.

In Table 4, first, compared with the ambient temperature (20 °C), the color of all specimens began to change to grayish-white as the exposure temperature increased, and finally (900 °C) became yellow, which was due to the loss of water of lower temperatures and the chemical decomposition of higher temperatures [12,13,14]. In addition, with the increase in the black biochar content, the color of the specimen turned black, but at 900 °C, CC, CB2, and CB5 became a uniform yellow. In addition, no obvious explosive spalling occurred in any specimens. With the increase in exposure temperature, the width and number of cracks increased, which was confirmed by many previous studies [15]. The main cause of the generation of cracks was the internal pore pressure caused by the evaporation of free water and the dehydration of hydration products. Furthermore, as the content of biochar increased, the width and number of cracks decreased, potentially due to the pores of the biochar contributing to the release of vapor pressure.

### 3.2. Macro Weight Loss

The weight loss percentages of CC, CB2 and CB5 exposed to different temperatures are provided in Table 5. As shown in Table 5, the following results were obtained: (1) The weight loss before 300 °C includes the evaporation of free water and the dehydration of hydration products AFt (Ettringite), AFm and CSH (Calcium-silicate-hydrate) [16]. As shown in Table 5, the weight loss percentage of 20–300 °C increased with increasing biochar content. This indicates that biochar contributes to the release of free water and bound water, which may be related to the porous structure of biochar. (2) The weight loss percentage of the specimen exposed to 300–550 °C decreased with the increase in the biochar content. The weight loss at 300–550 °C was mainly the dehydration of CH (Calcium hydroxide) [17,18], and the CH content of CB2 and CB5 decreased with the addition of biochar [19]. (3) The weight loss percentage with exposure to 550–900 °C increased with the increase in the biochar content. In this temperature range, the weight loss of the specimen mainly included the decomposition of the hydration product CSH [20,21] and the ignition loss of biochar. From the results in Table 4, we did not observe the black biochar in the CB2 and CB5 specimens at 900 °C. Therefore, we consider that the weight loss in the temperature range of 550–900 °C includes the ignition loss of a large amount of biochar. Biochar is a carbon-rich product of pyrolysis under anaerobic conditions. The carbon in biochar reacts to generate CO_2_ gas and escapes through the pores and cracks of the specimen when exposed to high temperatures and oxygen. Thus, the increase in the weight loss percentage at 550–900 °C with the increase in biochar content is mainly related to the ignition loss of biochar.

### 3.3. Residual Compressive Strength

**Figure 3:**
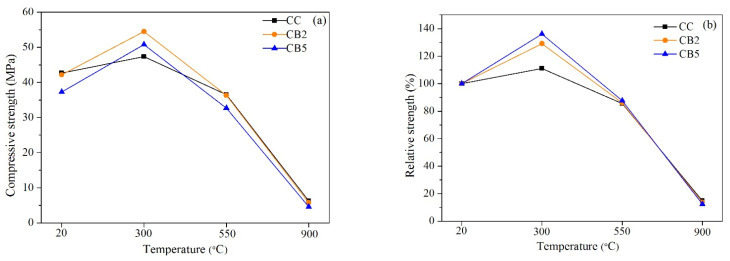
(**a**) Compressive strength of CC, CB2, and CB5 specimens at 20 °C and the residual compressive strength when exposed to 300, 550, and 900 °C. (**b**) Relative compressive strength of CC, CB2, and CB5 normalized to the strength at 20 °C.

Figure 3 shows the compressive strength of all specimens exposed to different temperatures and the relative residual compressive strength relative to 20 °C. At 20 °C, the compressive strengths of CC, CB2 and CB5 were 42.7, 42.2 and 37.3 MPa, respectively. The compressive strength of CB2 (2%) was comparable to the control (CC). The compressive strength of CB5 (5%) was significantly reduced because excessive porous biochar adversely affects the strength [22].

For 300 °C, the residual compressive strengths of CC, CB2 and CB5 were 47.4, 54.5 and 50.8 MPa, respectively. The internal pore pressure generated by the evaporation of free water and the dehydration of hydration products in this temperature range had an adverse effect on the strength. However, the compressive strength of all specimens was greater than their compressive strength at 20 °C. This results from the positive effect of the internal autoclaving effect at 300 °C on the compressive strength being greater than the negative effect mentioned above [23]. In addition, as shown in Figure 3a, compared with CC, the compressive strength of CB2 and CB5 increased by 7.1 and 3.4 MPa, respectively. As shown in Figure 3b, as the content of biochar increased, the relative compressive strength at 300 °C increased. This indicates that biochar contributes to the development of the compressive strength of cementitious materials exposed to 300 °C, which may be mainly attributed to the pores of the biochar increasing the release of pore pressure, thereby reducing the number of cracks (Table 4).

For 550 °C, first, the residual compressive strengths of CC, CB2 and CB5 were 36.5, 36.3 and 32.7 MPa, respectively. These strengths are lower than the compressive strengths at 20 °C. As the temperature continued to increase to 550 °C, the hydration products CH decomposed, the width and number of cracks increased (Table 4), and the compressive strength decreased. We also found that CB2 and the control maintained comparable residual compressive strength at 550 °C, as shown in Figure 3a. Second, the relative residual strengths of CC, CB2 and CB5 were similar, with values of 85.5%, 86.0% and 87.6%, respectively, as shown in Figure 3b. After exposure to 550 °C, the residual strength of the specimen was still maintained at 85–88% of the strength of 20 °C. With the increase in biochar content, the relative residual compressive strength slightly increased, indicating that biochar still had a positive effect on the high-temperature resistance of the biochar-cementitious specimen at 550 °C.

For 900 °C, the residual strengths of CC, CB2 and CB5 were 6.3, 5.8 and 4.6 MPa, respectively. Compared to 550 °C, the strength decreased significantly. The relative residual strengths were 14.75%, 13.74% and 12.33%, respectively. Two factors mainly caused these significant strength reductions: (1) chemical factor: the hydration product CSH was completely converted into C_n_S [20]; (2) physical factor: as the temperature continued to increase, the cumulative cracks increased. In addition, as the content of biochar increased, the residual strength and relative strength slightly decreased, which is inconsistent with the cracks result (see Table 4, physical factor). This may be due to another physical factor causing the reduction in the residual strength of the biochar blend paste, i.e., the ignition loss of biochar may produce additional pores, which may impair the compressive strength.

### 3.4. UPV

**Figure 4:**
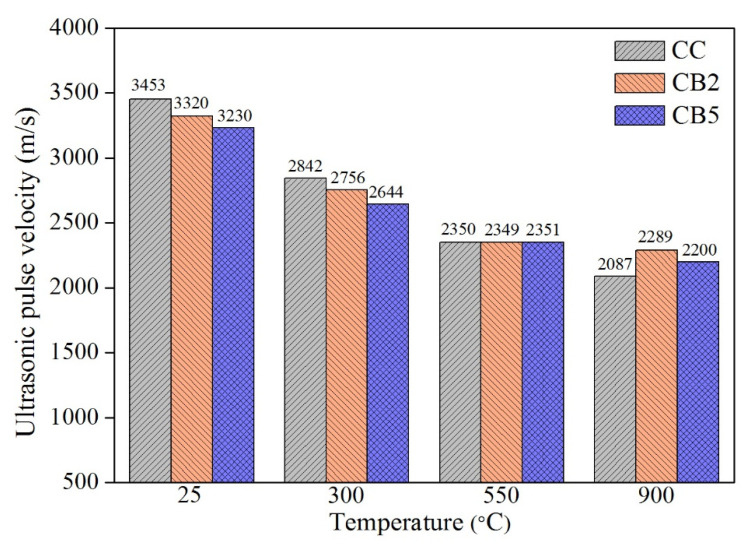
UPV of the paste specimens exposed to 20, 300, 550 and 900 °C.

Figure 4 shows the velocity of the ultrasonic pulses through the specimens exposed to different temperatures. UPV can be used to characterize the internal deterioration of the specimen at high temperatures.

For 20 °C, as shown in Figure 4, the UPV decreased with the increase in the biochar content. The internal pores of the biochar and entrapped pores due to the addition of biochar reduced the compactness of the sample [22], thereby slowing the propagation in the ultrasonic pulse.

For 300 °C, compared with 20 °C, the large amount of free water evaporation and the generation of micro cracks led to a decrease in the UPV of all specimens [24]. In addition, UPV decreased with the increase in biochar content at 300 °C, which is inconsistent with the cracks and strength results. This finding may be because the pores of the biochar promoted the release of free water and bound water inside the specimen. This is consistent with the result of the weight loss of the specimen at 20–300 °C (Table 5).

As shown in Figure 4, at 550 °C, firstly, compared with 300 °C, the UPV of all specimens reduced, resulting in the further decomposition of hydration products and the reduced compactness of the specimens. In addition, the UPV of CC, CB2 and CB5 were comparable at 550 °C. This is the result of the combined effect of two factors: (1) the pores of the biochar had a negative impact on the development of UPV, which was mentioned above; (2) as the content of biochar increased, the apparent reduction in the cracks of the specimen at 550 °C (Table 4) had a positive effect on the development of UPV. At 550 °C, the two effects cancel each other out to produce a comparable UPV value.

At 900 °C, the UPVs of CB2 and CB5 were already greater than that of CC. Obviously, with the increase in accumulated cracks, cracks became the main factor affecting the UPV.

### 3.5. FTIR Analyses

**Figure 5:**
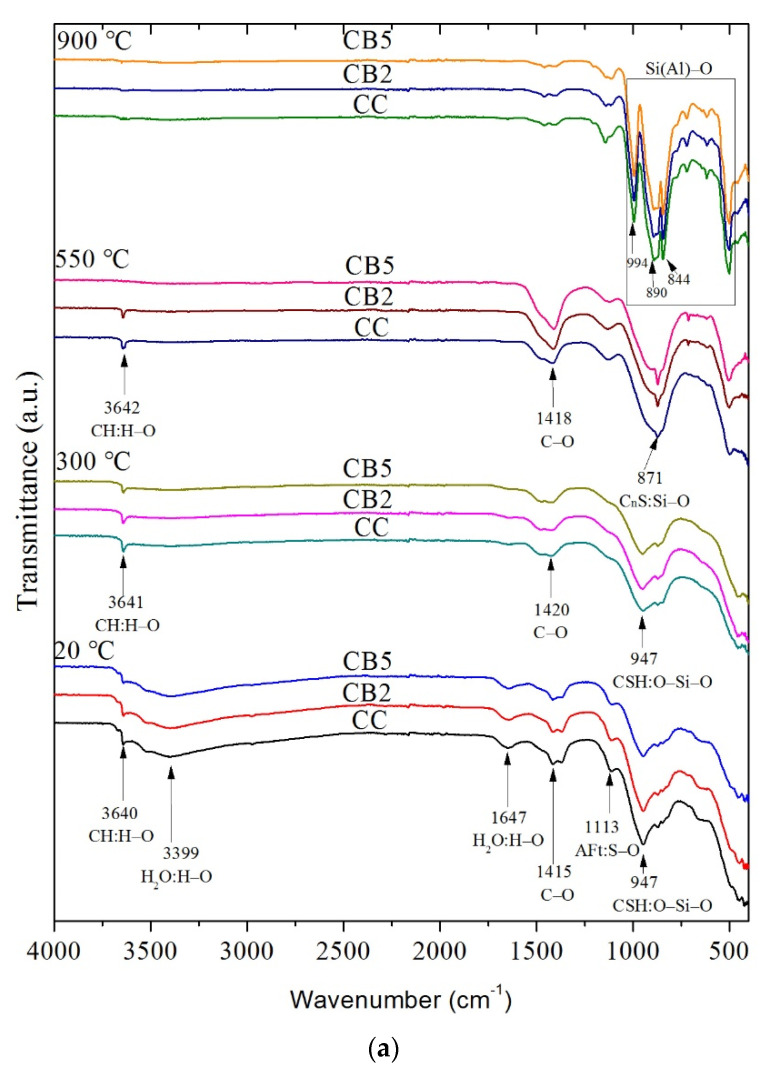

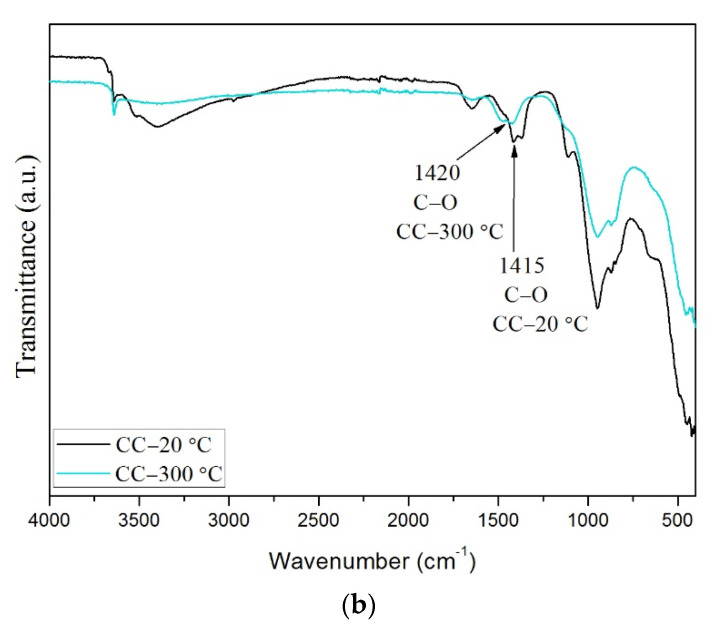
(**a**) FTIR spectra of specimens exposed to 20, 300, 550 and 900 °C. (**b**) Comparison of CC curves at 20 °C and 300 °C.

FTIR was used to characterize the functional groups of the products exposed to different temperatures. Figure 5a shows the FTIR spectra of CC, CB2 and CB5 exposed to 20, 300, 550 and 900 °C.

First, as shown in Figure 5a, regardless of exposure to any temperature (20, 300, 550 or 900 °C), compared with CC, CB2 and CB5, no new absorption peaks of functional groups or any absorption peak of the functional group disappearance occurred and there was no obvious position migration. This indicates that the biochar had not changed the types of functional groups of the product exposed to ambient or to high temperature.

Secondly, the absorption peak changes in the FTIR at different temperatures are as follows: At 20 °C, the absorption peak near 3640 cm^−1^ is the stretching vibration of H-O in CH. The absorption peak at 947 cm^−1^ is the stretching vibration of O-Si-O of CSH. The peak at 1113 cm^−1^ is the stretching vibration of SO_4_^2-^ in AFt [25,26]. In addition, as the content of biochar increased, the peaks of CH, CSH and AFt showed a slight weakening trend.

Based on the comparison of the results between 20 and 300 °C, the following results were obtained: At 20 °C, the two absorption peaks near 3399 and 1647 cm^−1^ represent the vibration of H-O in free water, and at 300 °C, these two peaks disappeared. At 20 °C, the absorption peak near 1415 cm^−1^ is the asymmetrical stretching vibration of C-O in Mc, and at 300 °C, it changed to a higher band (1420 cm^−1^) and the intensity decreased, indicating that Mc decomposed [21], as showed in Figure 5b. At 300 °C, the SO_4_^2-^ peak of AFt disappeared, indicating that AFt decomposed.

For 550 °C, the peak of H-O at 3640 cm^−1^ changed to a higher band (3642 cm^−1^) and weakened (CC and CB2) or disappeared (CB5), as a result of CH decomposition. The weak peak of CH in CC and CB2 at 550 °C may have been caused by the rehydration of CaO in the specimen preparation process [16]. The peak at 1418 cm^−1^ is the vibration of C-O in CaCO_3_, which may be the CaCO_3_ formed by the reaction of CaO with CO_2_ during the cooling process or preparation of the specimen [27]. In addition, a new peak was generated at 550 °C: the absorption peak near 871 cm^−1^ was caused by the vibration of Si-O in C_n_S due to the continuous decomposition of CSH into C_n_S.

For 900 °C, the absorption peaks at 994, 890, and 844 cm^−1^ were the vibrations of Si(Al)-O in the products of complete decomposition of CS(A)H [21,28].

Finally, as shown in Figure 5a, regardless of exposure to any high temperature, no obvious peak intensity weakening or enhancement was captured as the biochar content increased, which may be attributed to the lower biochar content.

### 3.6. XRD Analyses

**Figure 6:**
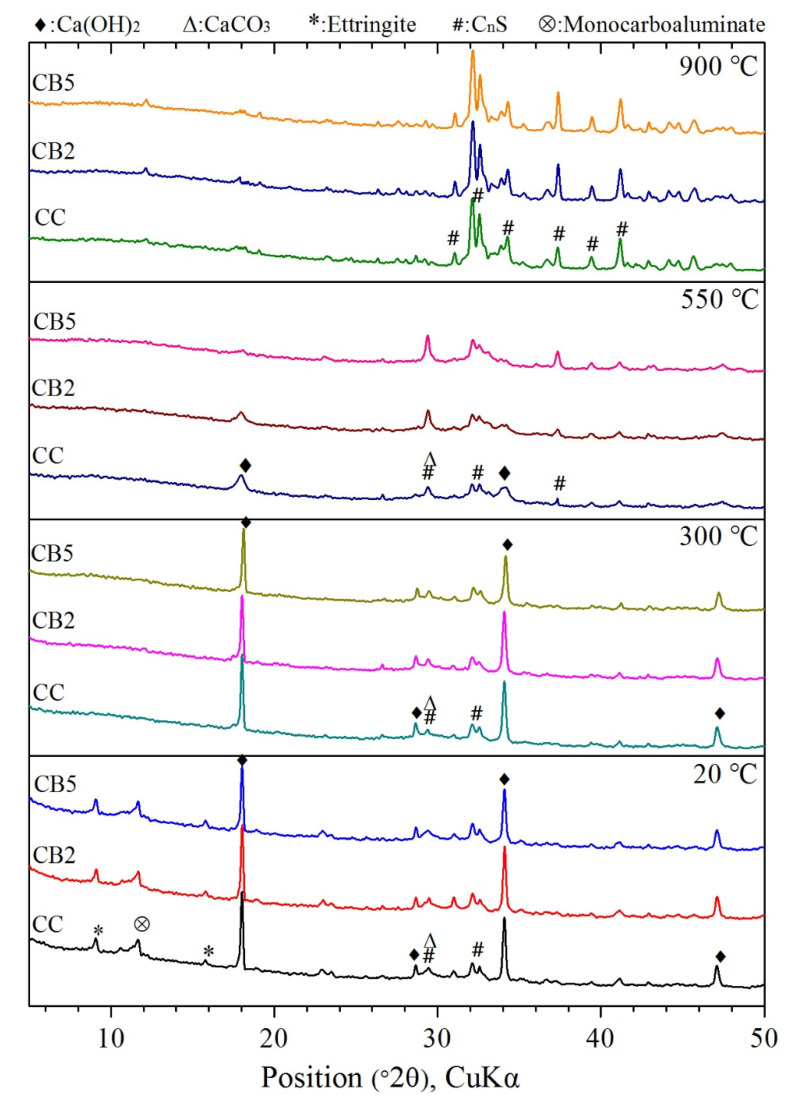
XRD analysis of specimens exposed to 20, 300, 550 and 900 °C.

XRD was used to characterize the crystalline phase components of the products exposed to different temperatures. Figure 6 summarizes the XRD curves of all specimens exposed to 20, 300, 550 and 900 °C. As shown in Figure 6, first, compared with CC, regardless of exposure to any high temperature, CB2 and CB5 showed no new characteristic peaks of product appearance or any characteristic peak disappearance. This indicated that the types of the crystalline components in the product of the biochar mixed paste exposed to high temperatures did not change with the addition of biochar.

In Figure 6, at 20 °C, compared with CC, as the content of biochar increases, the main characteristic peaks of CH near 18° and 34.1° weaken, especially the peak of CH in CB5 shows a significant weakening trend. This indicates that the addition of biochar inhibited the formation of hydration products [19].

For 300 °C, the peaks of AFt located about 9° and 16° at 20 °C disappeared at 300 °C, and the characteristic peaks of Mc near 12° also disappeared. These results indicate that AFt and Mc decomposed when exposed to 300 °C. These results are consistent with the FTIR results.

For 550 °C, compared with 300 °C, the peaks of CH located at different positions disappeared (CB5) or their intensity significantly decreased (CC and CB2), related to the decomposition of CH in the temperature range of 300–550 °C. The increase in the intensity of the peak near 29.3° was due to CaCO_3_ production [29]. These results are consistent with the results of FTIR.

For 900 °C, compared with exposure to other temperatures, all the characteristic peaks of the hydration products disappeared. As the temperature continued to increase to 900 °C, the products CSH completely decomposed to C_n_S. Thus, only the peaks of C_n_S crystal peaks were captured. With the increase in biochar content, no obvious changes in the intensity of the C_n_S peak were observed.

### 3.7. TG Analyses

**Figure 7:**
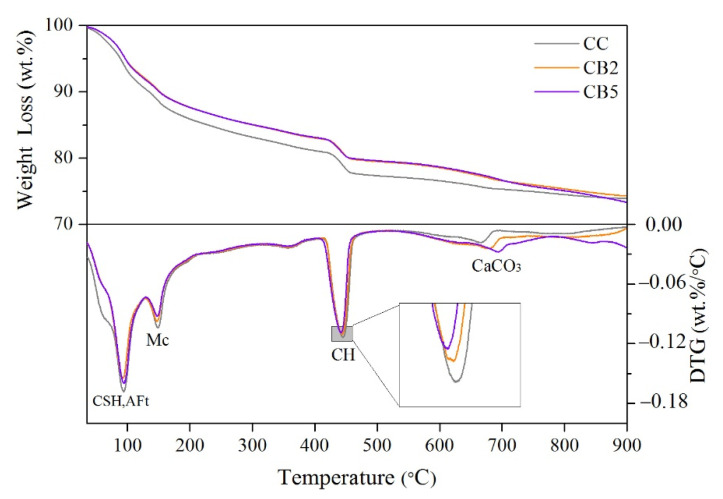
TG and DTG curves of specimens from 20 to 900 °C.

Figure 7 shows the TG and derivative thermogravimetry (DTG) curves of CC, CB2, and CB5; Table 6 lists the percentage of weight loss for different temperature ranges obtained through the TG test. As shown by the DTG curves in Figure 7, the weight loss at 20–300 °C includes the decomposition of CSH, AFt, and Mc, and the evaporation of free water. The weight loss at 300–550 °C represents the dehydroxylation of CH. The weight loss at 550–900 °C is mainly the decarburization of CaCO_3_ [30]. The sources of CaCO_3_ in the product can be the carbonation generated during the preparation of the specimens and the presence of small amounts of CaCO_3_ in the cement raw material [20,31,32]. Combined with Table 6, we found that as the content of biochar increased, the weight loss at 20–300 and 300–550 °C decreased, indicating that the hydration products (CSH, AFt, Mc, and CH) were reduced as a result of the inhibition effect caused by the addition of biochar [19].

### 3.8. SEM Analyses

**Figure 8:**
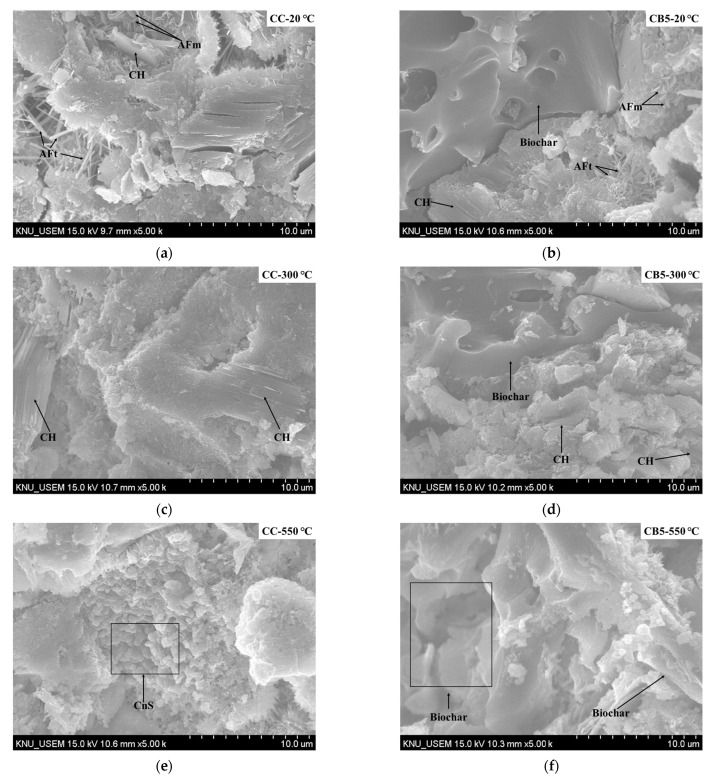

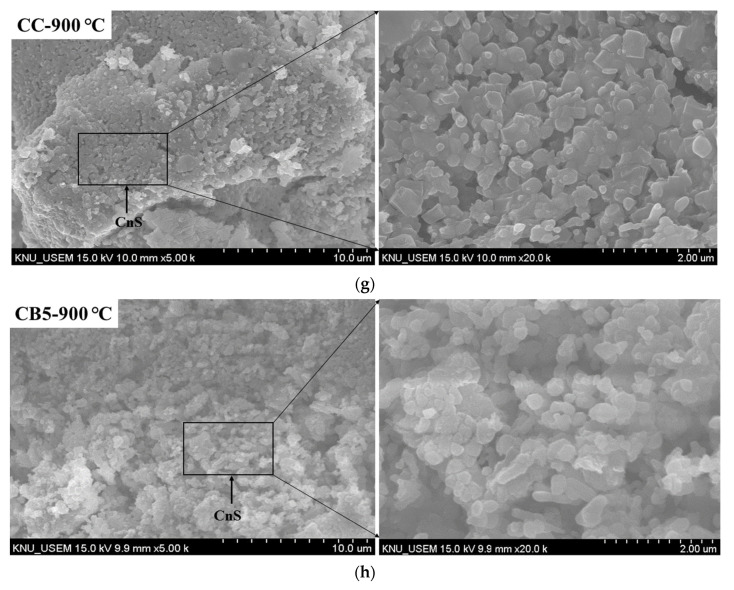
SEM images of CC and CB5 exposed to 20, 300, 550 and 900 °C. (**a**) SEM image of CC exposed to 20 °C. (**b**) SEM image of CB5 exposed to 20 °C. (**c**) SEM image of CC exposed to 300 °C. (**d**) SEM image of CB5 exposed to 300 °C. (**e**) SEM image of CC exposed to 550 °C. (**f**) SEM image of CB5 exposed to 550 °C. (**g**) SEM image of CC exposed to 900 °C. (**h**) SEM image of CB5 exposed to 900 °C.

Figure 8 compares the SEM images of CC and CB5 exposed to 20, 300, 550 and 900 °C. For 20 °C (Figure 8a,b), the hydration products CH (large hexagonal prism and large prismatic crystals), AFt (needle-shaped crystals), and AFm (hexagonal plate crystals) can be clearly observed in samples CC and CB5. In addition, biochar with a porous morphology was captured in sample CB5.

For 300 °C (Figure 8c,d), CH was still observed in CC and CB5, and biochar was still observed in CB5. However, AFt and AFm were not observed. This is consistent with the XRD and TGA results, which further proves that the AFt and AFm in specimens exposed to 300 °C were decomposed. Comparing Figure 8a,c, at 300 °C, the microstructure of the paste was denser than at 20 °C. This may be due to the autoclaving effect, and this also explains why the compressive strength increased at 300 °C.

For 550 °C (Figure 8e,f), no CH was observed in CC and CB5, which is related to the decomposition of CH at 400–500 °C (Figure 7). The biochar in CB5 was still observed. In addition, some C_n_S was captured in CC, which is related to the continuous decomposition of CSH [15].

For 900 °C (Figure 8g,h), due to the complete decomposition of CSH, a large amount of C_n_S in CC and CB5 was observed. Moreover, it was difficult to observe porous biochar in CB5, indicating that the biochar was lost due to ignition.

## 4. Discussion

### 4.1. Relationship between the Macro Weight Loss and the Micro Weight Loss

Comparison of the relationship between macro weight (Table 5) and micro weight loss (Table 6) with increasing biochar content showed consistency (300–550 °C and 550–900 °C) or inconsistency (20–300 °C) in different temperature ranges, and the reasons for this result are discussed below according to the different ranges.

First, for 20–300 °C, the macro and micro weight losses showed inconsistent trends with increasing biochar content, i.e., the macro weight loss increased with increasing biochar content, whereas the micro weight loss showed the opposite trend. In addition, the macro weight loss percentage was much larger than the micro weight loss because the weight loss of macro specimens (50 × 50 × 50 mm^3^) contained a large amount of free water loss in addition to bound water in this temperature range. The main reason for the increase in macro weight loss with increasing biochar replacement ratio was that the addition of biochar promoted the release of vapor generated by the conversion of free water. The trend of decreasing micro mass loss with increasing biochar replacement ratio was related to the decrease of hydration products (CSH, AFt, AFm) with increasing biochar content.

Secondly, for 300–550 °C, both weight loss trends are consistent, i.e., with the increase in biochar content, the weight loss decreases. Both weight losses were mainly due to the decomposition of CH. In addition, the macro weight loss was lower than the micro weight loss, which may be related to the recrystallization of CaO in macro specimens during the cooling. In contrast, no recrystallization of CaO occurred in the micro powder samples under a nitrogen environment.

Finally, for 550–900 °C, both weight loss trends were consistent with an increase in biochar content. The macro weight loss was mainly due to the continuous decomposition of CSH and the ignition loss of biochar in the oxygen environment. The micro weight loss was mainly due to the continuous decomposition of CSH and the decomposition of the small amount of CaCO_3_ generated during the specimen preparation. The weight loss of trace CaCO_3_ in the cement raw material was present in both macro and micro.

### 4.2. Relationship between Residual Strength and Cracks

As shown in Table 4 and Figure 3a, first, as the temperature increased, the number and width of cracks increased in all specimens, and the residual compressive strength first (300 °C) increased and then (550 and 900 °C) decreased. Secondly, as the content of biochar increased, the number and width of cracks decreased when exposed to any temperature, the residual compressive strength at 300 °C increased, and the residual compressive strength at 550 and 900 °C slightly decreased. In short, we observed no uniform trend for the changes in the residual strength and cracks. This is explained by the residual compressive strength being influenced by a combination of physical factors, such as crack widths and number and additional pores due to the ignition loss of biochar, and chemical factors, such as product transformation at high temperatures.

### 4.3. Relationship between Compressive Strength and UPV

As shown in Figure 3a and Figure 4, for 20 °C, the compressive strength and UPV showed a positive correlation, i.e., as the compressive strength decreased (with increasing biochar content), the UPV decreased, which is consistent with the findings of many previous studies [33]. For 300, 550 and 900 °C, the residual compressive strength and UPV did not show a uniform trend. Normally, the strength and UPV of cement-based materials are positively correlated, but this is obviously not applicable to biochar-blended paste specimens exposed to high temperatures. The addition of biochar had a negative effect on the propagation of ultrasonic waves inside the specimens exposed to high temperatures, i.e., the pores of the biochar slow the propagation of ultrasound pulses and promote the evaporation of free water, thus reducing the UPV. In contrast, it has a positive effect on the propagation of ultrasonic waves, i.e., the pores of biochar reduce the pore pressure when exposed to high temperatures, reducing the generation of cracks, thus increasing the UPV. The two mechanisms together act on the uncertainty in the development of UPV and the residual compressive strength trends of biochar mixed paste specimens.

The relationships of the various properties indicate that it is more comprehensive and necessary to jointly characterize the high-temperature resistance of biochar cementitious materials using various methods.

## 5. Conclusions

Various methods (optical microscopic images, weight loss, residual strength and UPV) were used to compare the meso cracks and macro characteristics of the control and the biochar mixed paste specimens exposed to different temperatures (20, 300, 550 and 900 °C). Microscopic characterization was achieved using multiple methods (TG, XRD, FTIR and SEM). The connection between the various results was discussed. The following conclusions regarding the high-temperature resistance of biochar cementitious composites were obtained:

1. The cracks’ widths and number of specimens exposed to any high temperature (300, 550 or 900 °C) decreased with increasing biochar content. The internal pores of biochar promoted the release of vapor pressure, which reduced the generation of cracks.
2. The macro weight loss percentage of specimens exposed to 20–300 °C increased with the biochar content because biochar contributed to the release of free water and bound water. The macro weight loss percentage of the specimen exposed to 300–550 °C decreased with the increase in the biochar content, related to the decrease in CH. The macro weight loss percentage of the specimen exposed to 550–900 °C increased with the increase in the biochar content, potentially related to the ignition loss of biochar.
3. From 20 to 300 °C, the strength of all specimens increased. Moreover, the strength increase of the biochar-blended specimen was greater than that of the control specimen. First, at 300 °C, the residual compressive strength of specimens increased by 7.1 and 3.4 MPa with 2% and 5% of biochar added compared to no biochar added. Second, at 550 °C, the residual compressive strengths of the control and specimens with 2% biochar were comparable. Third, at 900 °C, the residual compressive strength slightly decreased with the increase in biochar content. Summarily, due to the internal pores of biochar, the addition of an appropriate amount of biochar can improve the high temperature mechanical properties.
4. From 20 to 300 °C, the UPV of all specimens decreased. For 300 °C, the UPV of the specimen decreased with the increase in biochar content. At 550 °C, the UPVs of the control and specimens with 2% and 5% biochar were comparable. For 900 °C, the UPV of the specimens with 2% and 5% biochar was greater than that of the control as a result of the reductions in meso cracks.
5. FTIR and XRD results indicated that biochar does not change the types of functional groups and crystalline phases of the products of cementitious materials exposed to high temperatures. The free water, AFt, and AFm decomposed before 300 °C. Most of the CH peaks disappeared at 550 °C, and a small amount of recrystallized CH peaks were present. Only the C_n_S peaks were present at 900 °C.
6. TG analysis showed that as the content of biochar increased, the weight loss at 20–300 and 300–550 °C decreased, indicating that the hydration products (CSH, AFt, Mc, and CH) were reduced.
7. The SEM images showed that AFt and AFm disappeared at 300 °C. CH disappeared at 550 °C. Biochar particles were difficult to observe at 900 °C and a large amount of C_n_S was observed at 900 °C.
8. The relationships of various properties indicated that it is more comprehensive and necessary to jointly characterize the high-temperature resistance of biochar cementitious materials using various methods. Such as the simultaneous consideration of the trends in the meso cracks, UPV, and residual compressive strength indicates that meso cracks and biochar pores play an important role in influencing the high-temperature-resistance performance of biochar–cement composites. In addition, the experimental FTIR findings at various high temperatures agree with the XRD, TG and SEM results.

In summary, as a porous material, biochar can reduce the internal vapor pressure of cementitious composites at high temperatures and improve the high-temperature resistance of concrete. Macro, meso and micro synergistic analyses are necessary to elucidate the mechanism of the high-temperature resistance of biochar-blended cementitious composites. The solution to improve the high temperature performance of cement composites is to replace the cement with biochar by an appropriate amount of addition (e.g., 2wt% replacement cement).

## Figures and Tables

**Table 1:** Chemical compositions of biochar and cement.

Chemical Composition (%)	Cement	Biochar
CaO	63.90	0.23
SiO_2_	21.29	19.17
Al_2_O_3_	5.33	0.04
SO_3_	2.57	0.14
MgO	2.06	0.10
P_2_O_5_	0.09	0.15
Fe_2_O_3_	2.74	0.05
K_2_O	1.08	1.19
TiO_2_	0.23	-
ZnO	0.11	-
CO_2_	-	78.74
MnO	-	0.04
Cl	-	0.10
Na_2_O	-	0.05
LOI	0.59	-

**Table 2:** Mix proportion of samples (mass %).

Sample	Cement	Biochar	Water	Water/Binder Ratio
CC (control)	100	0	50	0.5
CB2 (2%)	98	2	50	0.5
CB5 (5%)	95	5	50	0.5

**Table 3:** Experimental items and specimen types.

No.	Experimental Items	Specimen Type	Temperature (°C)
1	Optical Microscopic image	All samples	20, 300, 550 and 900
2	Macro weight loss	All samples	20–300, 300–550, 550–900
3	Compressive strength	All samples	20, 300, 550 and 900
4	UPV	All samples	20, 300, 550 and 900
5	FTIR	All samples	20, 300, 550 and 900
6	XRD	All samples	20, 300, 550 and 900
7	TGA	All samples	20–900
8	SEM	CC, CB5	20, 300, 550 and 900

**Table 4:** Optical microscopic images of the sample surface at 20, 300, 550 and 900 °C at 61× magnification.

Temperature	CC	CB2	CB5
20 °C	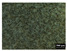	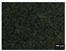	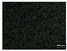
300 °C	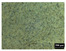	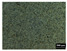	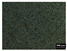
550 °C	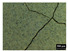	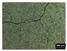	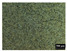
900 °C	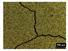	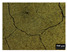	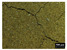

**Table 5:** Macro weight loss percentages of CC, CB2 and CB5 exposed to different temperatures.

Sample	Macro Weight Loss (%)		
	20–300 °C	300–550 °C	550–900 °C
CC	24.6	6.0	2.8
CB2	25.3	5.1	4.3
CB5	25.8	4.0	6.5

**Table 6:** TG weight loss percentages of CC, CB2 and CB5.

Sample	TG Weight Loss (%)		
	20–300 °C	300–550 °C	550–900 °C
CC	16.8	6.2	3.1
CB2	15.0	5.9	4.8
CB5	14.9	5.8	5.9

## Data Availability

The data presented in this study are available from the corresponding author upon reasonable request.
